# Supplementary material for: Modeling glioblastoma heterogeneity as a dynamic network of cell states
Source: Mol Syst Biol. 2021 Sep 16;17(9):e10105. doi: 10.15252/msb.202010105 (PMC8444284; doi:10.15252/msb.202010105)
Supplement: Supplementary file 6 — Source Data for Figure 5 [file MSB-17-e10105-s004.zip › Figure5A_sourcedata/GSEA_3071/nosigdb_stateII.GseaPreranked.1622795151171/LARSSON_STATE_6.html]

Details for gene set LARSSON\_STATE\_6[GSEA]

|  || Dataset | state23071 |
| Phenotype | NoPhenotypeAvailable |
| Upregulated in class | na\_neg |
| GeneSet | LARSSON\_STATE\_6 |
| Enrichment Score (ES) | -0.6929843 |
| Normalized Enrichment Score (NES) | -3.65703 |
| Nominal p-value | 0.0 |
| FDR q-value | 0.0 |
| FWER p-Value | 0.0 |
Table: GSEA Results Summary

  

Fig 1: Enrichment plot: LARSSON\_STATE\_6      
 Profile of the Running ES Score & Positions of GeneSet Members on the Rank Ordered List

  

| PROBE | GENE SYMBOL | GENE\_TITLE | RANK IN GENE LIST | RANK METRIC SCORE | RUNNING ES | CORE ENRICHMENT || 1 | HOPX |  |  | 768 | 0.510 | -0.1823 | No |
| 2 | ID1 |  |  | 769 | 0.509 | -0.1738 | No |
| 3 | EFTUD2 |  |  | 1795 | 0.299 | -0.4234 | No |
| 4 | TXNIP |  |  | 1918 | 0.283 | -0.4490 | No |
| 5 | PFDN5 |  |  | 2602 | -0.301 | -0.6136 | No |
| 6 | FTH1 |  |  | 2672 | -0.312 | -0.6256 | No |
| 7 | SRP14 |  |  | 2796 | -0.335 | -0.6506 | No |
| 8 | TSC22D4 |  |  | 2847 | -0.344 | -0.6573 | No |
| 9 | ATP6V1G1 |  |  | 2968 | -0.367 | -0.6809 | No |
| 10 | SERF2 |  |  | 2975 | -0.368 | -0.6763 | No |
| 11 | SNX3 |  |  | 2987 | -0.370 | -0.6729 | No |
| 12 | FAM20C |  |  | 3060 | -0.388 | -0.6843 | No |
| 13 | CITED1 |  |  | 3096 | -0.397 | -0.6864 | Yes |
| 14 | FKBP8 |  |  | 3097 | -0.398 | -0.6797 | Yes |
| 15 | ID3 |  |  | 3112 | -0.402 | -0.6765 | Yes |
| 16 | HEY1 |  |  | 3130 | -0.408 | -0.6739 | Yes |
| 17 | PLEKHB1 |  |  | 3131 | -0.409 | -0.6671 | Yes |
| 18 | CTNNA1 |  |  | 3163 | -0.418 | -0.6679 | Yes |
| 19 | COMMD6 |  |  | 3174 | -0.421 | -0.6633 | Yes |
| 20 | TOMM7 |  |  | 3198 | -0.428 | -0.6619 | Yes |
| 21 | SMDT1 |  |  | 3228 | -0.435 | -0.6619 | Yes |
| 22 | SOX9 |  |  | 3230 | -0.435 | -0.6549 | Yes |
| 23 | MARCH2 |  |  | 3241 | -0.438 | -0.6501 | Yes |
| 24 | BRI3 |  |  | 3242 | -0.438 | -0.6428 | Yes |
| 25 | FYN |  |  | 3262 | -0.444 | -0.6401 | Yes |
| 26 | SKP1 |  |  | 3283 | -0.451 | -0.6375 | Yes |
| 27 | MARCKS |  |  | 3369 | -0.487 | -0.6506 | Yes |
| 28 | ZMAT3 |  |  | 3380 | -0.490 | -0.6449 | Yes |
| 29 | COL4A3BP |  |  | 3414 | -0.505 | -0.6447 | Yes |
| 30 | LAMTOR4 |  |  | 3463 | -0.524 | -0.6479 | Yes |
| 31 | QKI |  |  | 3467 | -0.526 | -0.6398 | Yes |
| 32 | METRN |  |  | 3468 | -0.526 | -0.6311 | Yes |
| 33 | MAP1LC3A |  |  | 3501 | -0.542 | -0.6300 | Yes |
| 34 | FABP5 |  |  | 3513 | -0.549 | -0.6236 | Yes |
| 35 | TUBB2A |  |  | 3554 | -0.572 | -0.6240 | Yes |
| 36 | RPS11 |  |  | 3560 | -0.577 | -0.6156 | Yes |
| 37 | BEX3 |  |  | 3575 | -0.587 | -0.6093 | Yes |
| 38 | FOXO3 |  |  | 3594 | -0.601 | -0.6038 | Yes |
| 39 | RTL8C |  |  | 3636 | -0.633 | -0.6034 | Yes |
| 40 | C1orf122 |  |  | 3638 | -0.634 | -0.5931 | Yes |
| 41 | SAT1 |  |  | 3659 | -0.653 | -0.5872 | Yes |
| 42 | SH3BGRL |  |  | 3663 | -0.654 | -0.5770 | Yes |
| 43 | ID2 |  |  | 3664 | -0.654 | -0.5662 | Yes |
| 44 | TNFRSF21 |  |  | 3669 | -0.660 | -0.5562 | Yes |
| 45 | OLFM2 |  |  | 3690 | -0.672 | -0.5499 | Yes |
| 46 | RAB31 |  |  | 3757 | -0.737 | -0.5540 | Yes |
| 47 | GABARAPL2 |  |  | 3768 | -0.759 | -0.5439 | Yes |
| 48 | RND2 |  |  | 3780 | -0.771 | -0.5338 | Yes |
| 49 | PTMS |  |  | 3791 | -0.780 | -0.5233 | Yes |
| 50 | SOX4 |  |  | 3797 | -0.786 | -0.5114 | Yes |
| 51 | FEZ1 |  |  | 3806 | -0.794 | -0.5002 | Yes |
| 52 | C1QL1 |  |  | 3828 | -0.819 | -0.4917 | Yes |
| 53 | C9orf16 |  |  | 3833 | -0.824 | -0.4790 | Yes |
| 54 | PFN2 |  |  | 3841 | -0.832 | -0.4669 | Yes |
| 55 | GNAI2 |  |  | 3845 | -0.835 | -0.4537 | Yes |
| 56 | MARCKSL1 |  |  | 3852 | -0.845 | -0.4411 | Yes |
| 57 | PBX1 |  |  | 3914 | -0.977 | -0.4400 | Yes |
| 58 | CTXN1 |  |  | 3932 | -1.022 | -0.4272 | Yes |
| 59 | NEAT1 |  |  | 3976 | -1.142 | -0.4189 | Yes |
| 60 | MXD4 |  |  | 3985 | -1.174 | -0.4013 | Yes |
| 61 | SESN3 |  |  | 4003 | -1.260 | -0.3846 | Yes |
| 62 | TUBA1A |  |  | 4006 | -1.302 | -0.3634 | Yes |
| 63 | NFIB |  |  | 4007 | -1.310 | -0.3416 | Yes |
| 64 | PNRC1 |  |  | 4022 | -1.397 | -0.3218 | Yes |
| 65 | ZNF428 |  |  | 4033 | -1.491 | -0.2994 | Yes |
| 66 | TUBB2B |  |  | 4063 | -1.810 | -0.2765 | Yes |
| 67 | CLU |  |  | 4069 | -1.908 | -0.2459 | Yes |
| 68 | BEX1 |  |  | 4072 | -2.034 | -0.2125 | Yes |
| 69 | GFAP |  |  | 4080 | -2.343 | -0.1753 | Yes |
| 70 | S100B |  |  | 4081 | -2.383 | -0.1356 | Yes |
| 71 | PMP2 |  |  | 4082 | -2.398 | -0.0956 | Yes |
| 72 | CRYAB |  |  | 4088 | -2.598 | -0.0536 | Yes |
| 73 | FABP7 |  |  | 4095 | -3.351 | 0.0007 | Yes |
Table: GSEA details [plain text format]

  

Fig 2: LARSSON\_STATE\_6: Random ES distribution      
 Gene set null distribution of ES for **LARSSON\_STATE\_6**
